# Supplementary figures and images for: In vivo recording of suprachiasmatic nucleus dynamics reveals a dominant role of arginine vasopressin neurons in circadian pacesetting
Source: PLoS Biol. 2023 Aug 29;21(8):e3002281. doi: 10.1371/journal.pbio.3002281 (PMC10465001; doi:10.1371/journal.pbio.3002281)

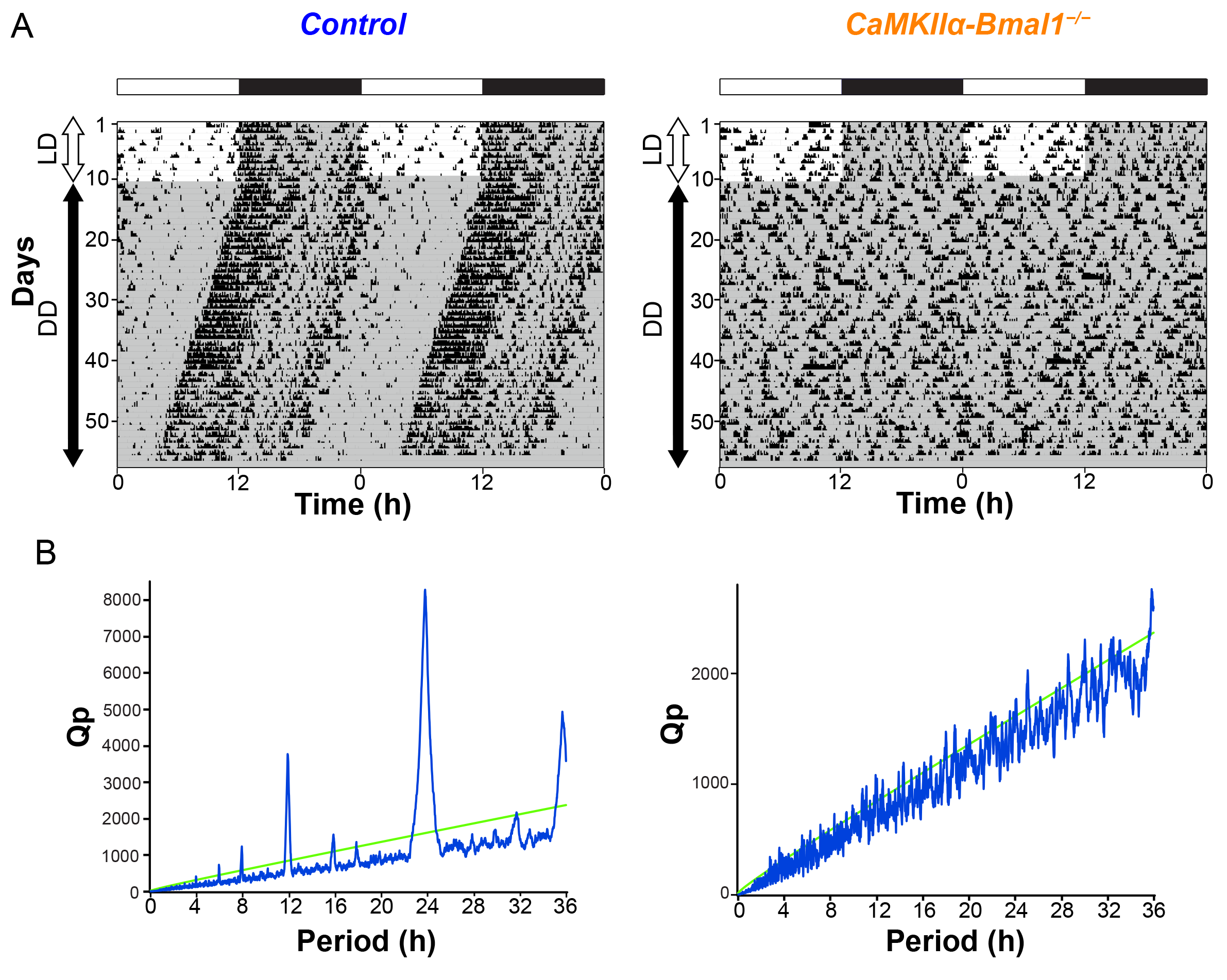

Supplement: S1 Fig — (A) Representative locomotor activity of control and CaMKIIα-Bmal1−/− mice (home-cage activity). Gray shading indicates the time when lights were off. (B) Representative periodograms of the locomotor activity rhythms of control (left) and CaMKIIα-Bmal1−/− mice (right) in DD. (TIF) [file pbio.3002281.s001.tif]

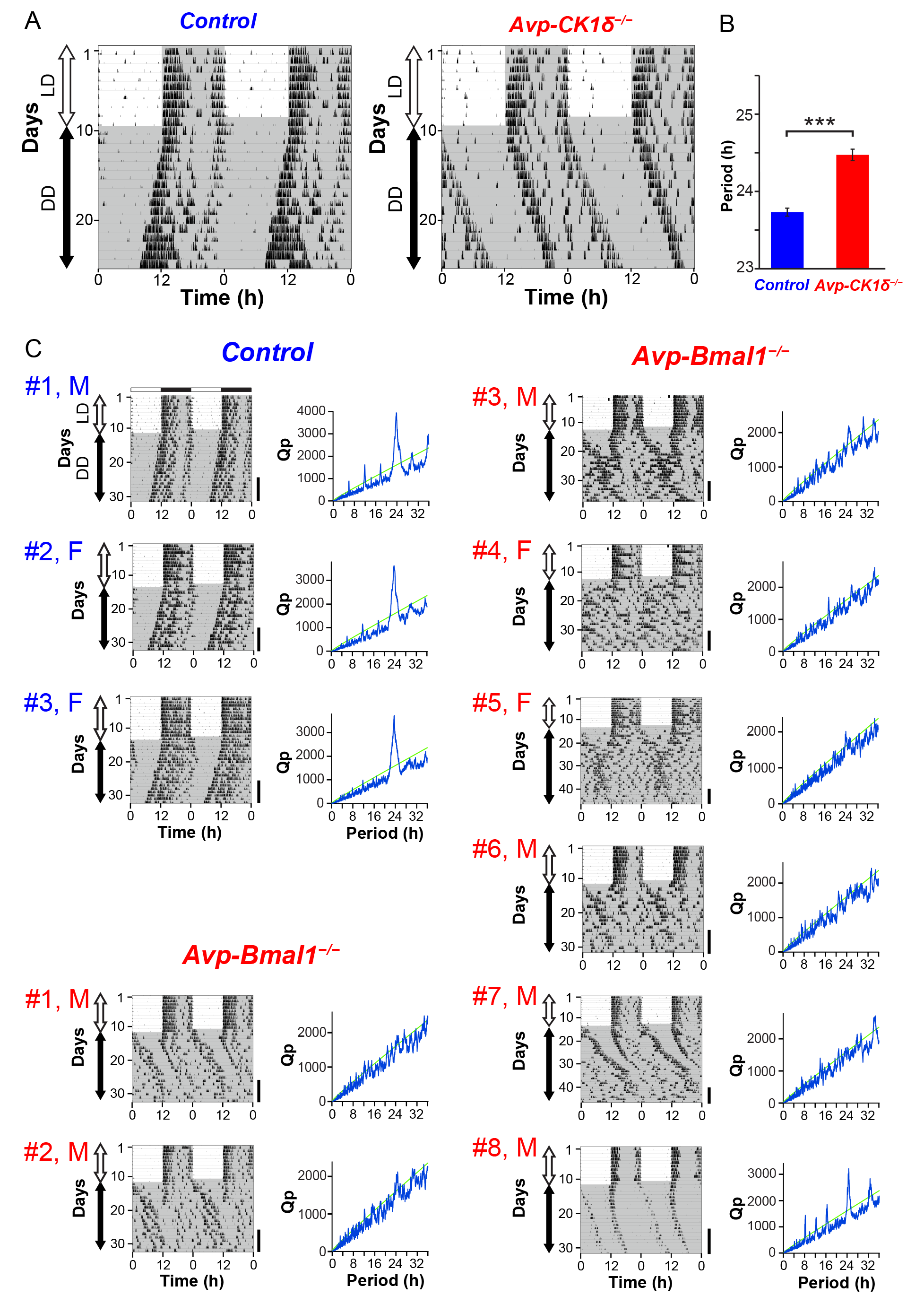

Supplement: S2 Fig — (A) Representative wheel-running activity of control and Avp-CK1δ−/− mice. Gray shading indicates the time when lights were off. (B) The free-running period of wheel-running activity in DD. Values are mean ± SEM; n = 4 for control, n = 5 for Avp-CK1δ−/− mice. ***P < 0.001 by two-tailed Student t test. (C) Left: Actograms of the wheel-running activity of 3 control and 8 Avp-Bmal1−/− mice (M, male; F, female). Gray shading indicates the time when lights were off. Right: Periodograms of the individual wheel-running activity rhythms in the last 7 days in DD (vertical black lines). Most Avp-Bmal1−/− mice were arrhythmic in the last part of recording in DD. (TIF) [file pbio.3002281.s002.tif]

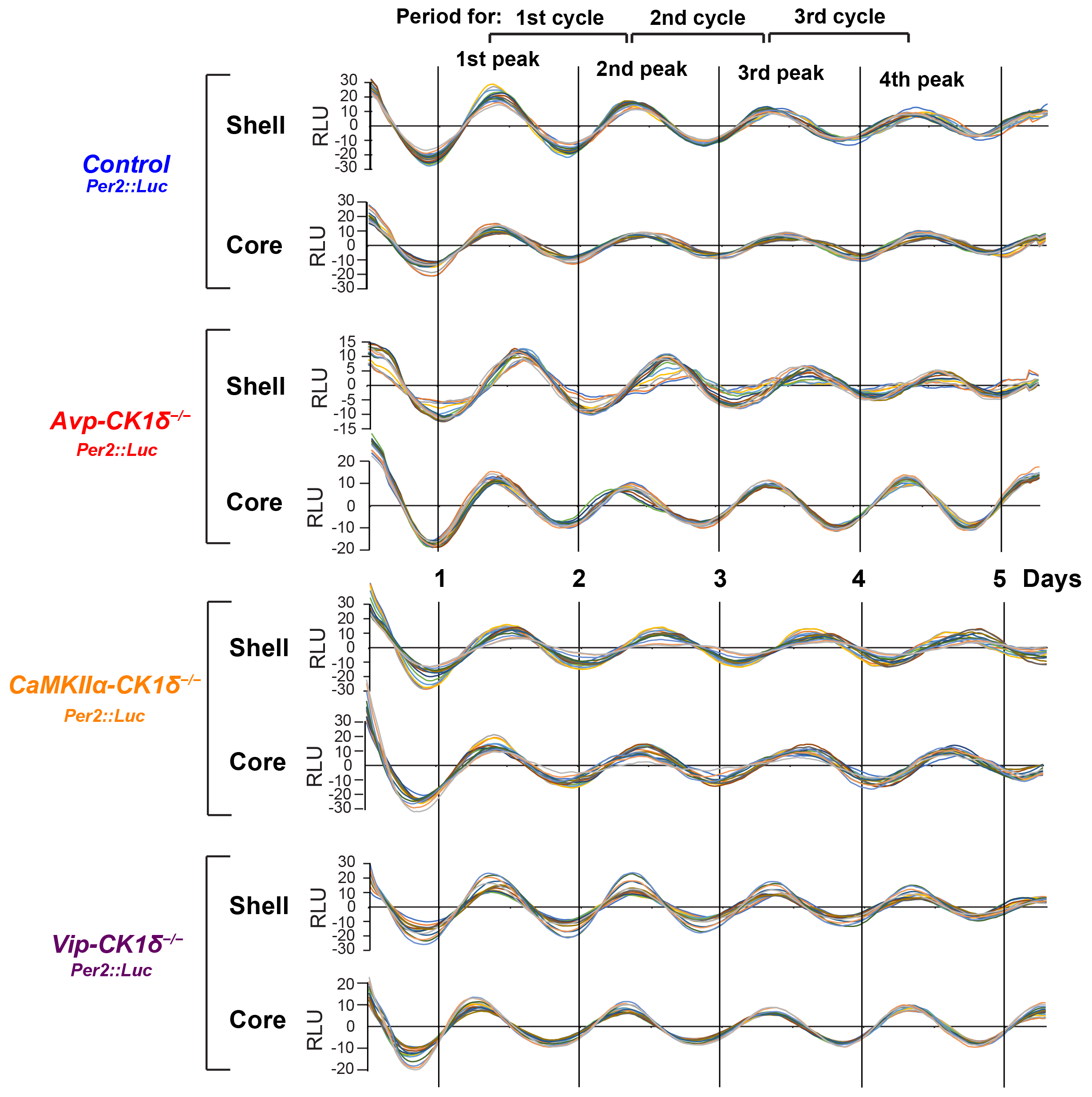

Supplement: S3 Fig — Representative bioluminescence data of 15 pixels in a row along the mediolateral axis within ROIs (15 × 15 pixels) are shown for each region and mouse line. All data were detrended, smoothed, and aligned at ZT12 on the day of slicing as starting points. Black vertical lines labeled day 1 indicate projected ZT0. These data were used for statistical analysis in Fig 3. (TIF) [file pbio.3002281.s003.tif]

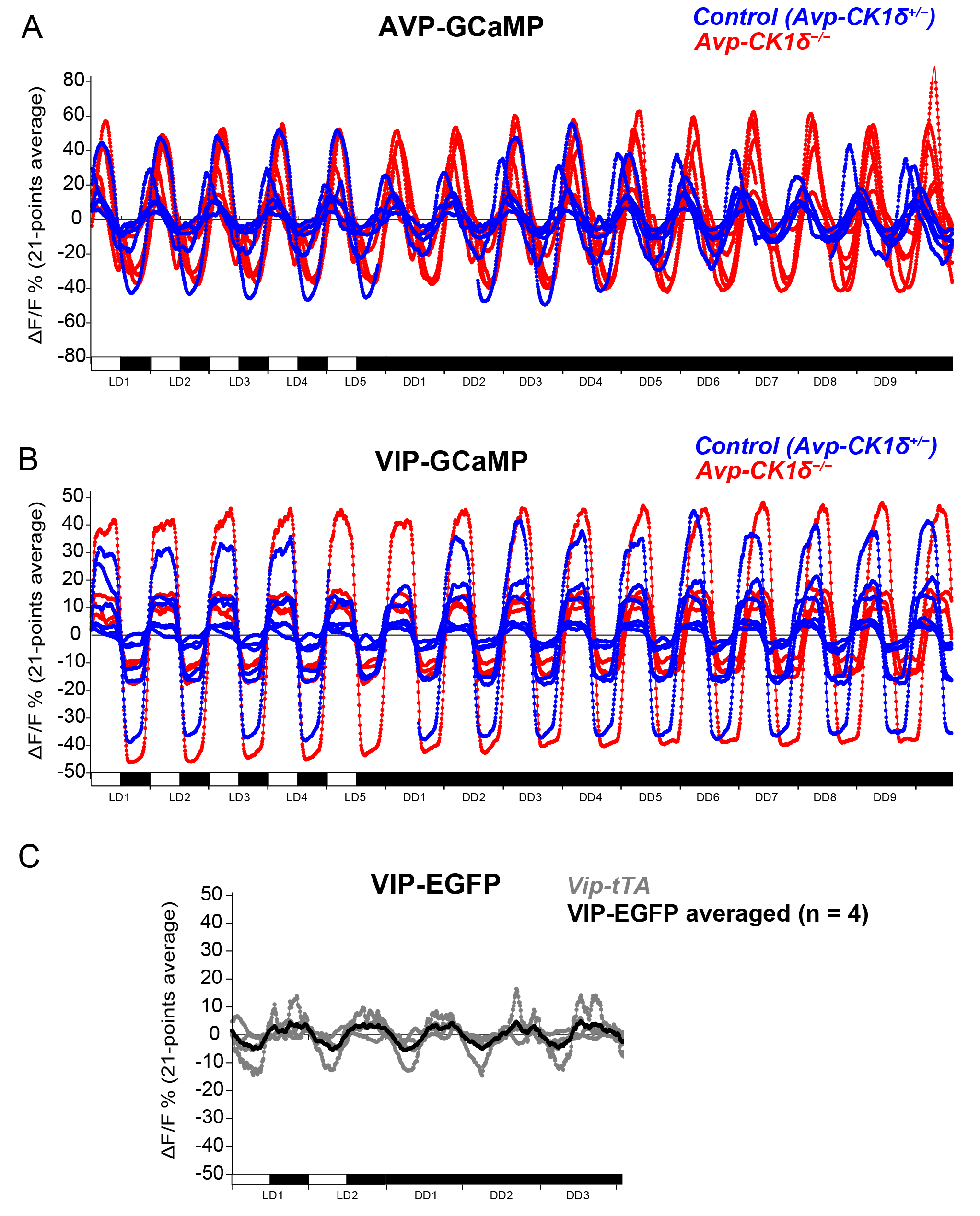

Supplement: S4 Fig — (A, B) Continuous recordings of GCaMP fluorescence from SCN AVP neurons (A) or VIP neurons (B) for 15 days (5 days in LD, 10 days in DD). Red, Avp-CK1δ−/− (n = 6, 4); blue, control (Avp-CK1δ+/−, i.e., Avp-Cre; CK1δwt/flox, n = 5, 6). (C) Continuous recordings of EGFP fluorescence from SCN VIP neurons for 5 days (2 days in LD, 3 days in DD). Gray, individual trace; black, average. (TIF) [file pbio.3002281.s004.tif]

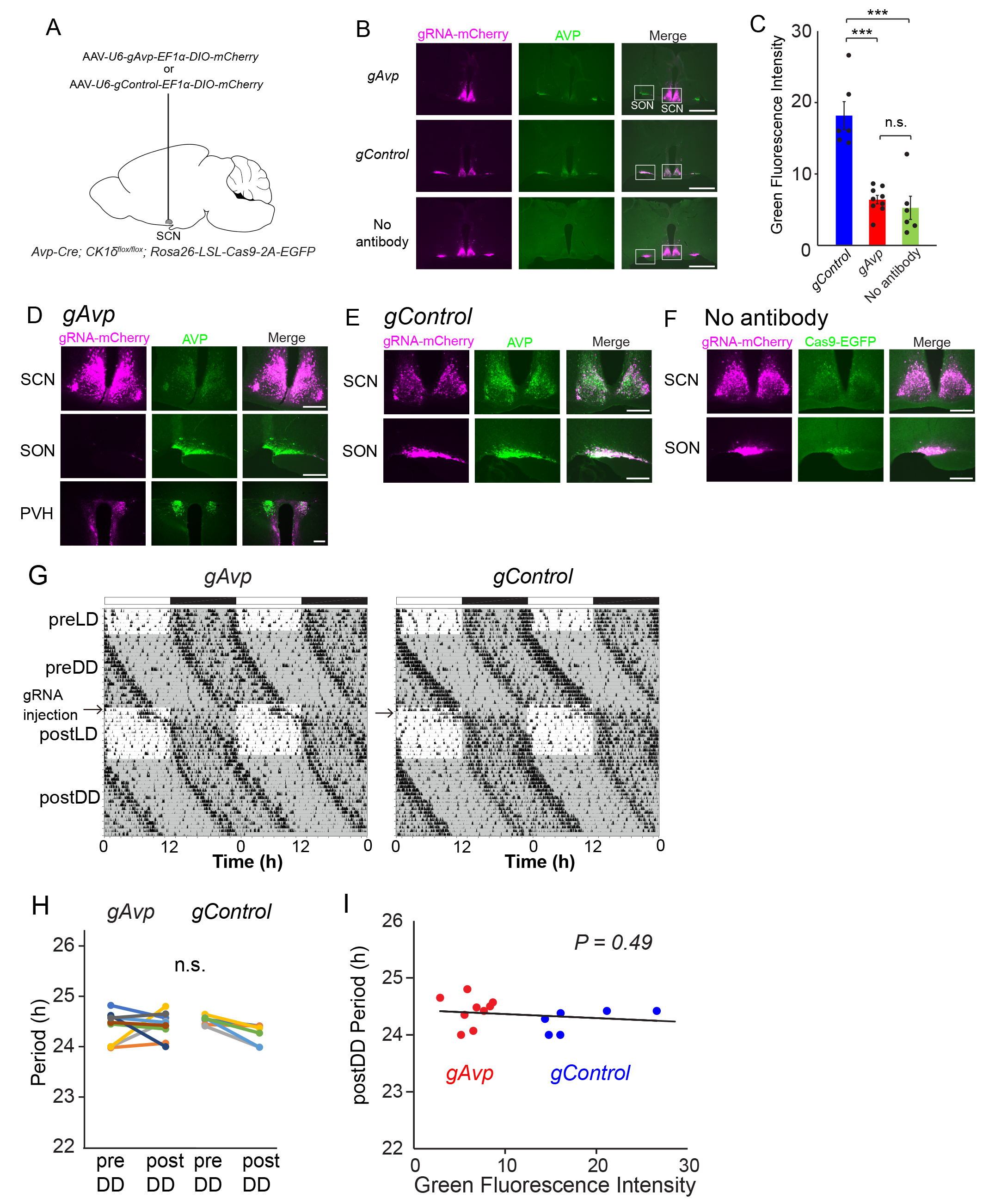

Supplement: S5 Fig — (A) Schematic diagram of viral vector (AAV-U6-gAVP-EF1α-DIO-mCherry or AAV-U6-gControl-EF1α-DIO-mCherry) injection at SCN in Avp-CK1δ−/− (Avp-Cre; CK1δ flox/flox; Rosa26-LSL-Cas9-2A-EGFP) mice. (B) Representative coronal slices with (gAvp) or without (gControl) Avp knockdown in the SCN stained with an anti-AVP antibody (green), showing reduced AVP immunoreactivity. Bottom slices (no antibody) are the coronal slices mock-stained without anti-AVP antibody to evaluate EGFP expression (green) derived from the Rosa26-LSL-Cas9-2A-EGFP allele. The white rectangles indicate the position of the enlarged images of (D-F). Scale bar, 1 mm. (C) Green fluorescence intensity of SCN slices immunostained (gAvp or gControl) or mock-stained (no antibody) for AVP. Note that the green fluorescence of immunostained slices is the sum of fluorescence derived from AVP immunoreactivity and EGFP. AVP expression is reduced to the background level in slices with Avp knockdown. n = 9 for gAvp, n = 6 for gControl, n = 6 for no antibody. ***P < 0.001 by one-way ANOVA with post hoc Ryan test. (D-F) Enlarged images of the SCN, supraoptic nucleus (SON), and the paraventricular nucleus of the hypothalamus (PVH) in gAvp (D), gControl (E), and no antibody (F) conditions. Scale bar, 200 μm. (G) Representative locomotor activity actograms of Avp-CK1δ−/− mice with (gAvp) or without (gControl) Avp knockdown in the SCN (home-cage activity). Before an AAV injection, mice were housed in LD for 1 w (preLD) and in DD for approximately 3 w (preDD). Subsequently, mice were turned back to LD condition and received a gAvp or gControl AAV injection surgery (arrows) and then were housed in LD for 2 w (postLD) and in DD for approximately 3 w (postDD). Gray shading indicates the time when lights were off. (H) Periods of locomotor activity in preDD (last 10 days) and postDD (last 10 days) with (gAvp) or without (gControl) Avp knockdown in Avp-CK1δ−/− mice. Each color indicates different mice. n = 9 for gAvp, n = 6 for gC [file pbio.3002281.s005.tif]
