# Supplementary material for: In vivo recording of suprachiasmatic nucleus dynamics reveals a dominant role of arginine vasopressin neurons in circadian pacesetting
Source: PLoS Biol. 2023 Aug 29;21(8):e3002281. doi: 10.1371/journal.pbio.3002281 (PMC10465001; doi:10.1371/journal.pbio.3002281)
Supplement: Attachment — Submitted filename: Response to Reviewers.pdf [file pbio.3002281.s008.pdf]

Response to Reviewers

The authors would like to thank all reviewers for their valuable comments and suggestions, which guided us in significantly improving our manuscript. Our detailed responses to the reviewers' concerns are itemized below.

Responses to Reviewer #2

One small point I think they could comment further on is the complexity of the genetic background of the mice. Circadian can vary with mouse strain (ie mice without mutations or genetic alterations) and as the authors already allude to, these studies used complex crosses and backgrounds. It is unclear just how close to a C57Bl6 background the mice models used in this study are. Some of the experimental variability reported in this manuscript could arise from comparing mice that are now deviating away from a 'wild-type' background.

Response: We thank the reviewer for the valuable suggestion. We have added a detailed description of the genetic background of all strains used in the study in the "Animals" section of the Materials and Methods.

"All lines were congenic on C57BL/6: *Avp-Cre* and *Vip-tTA* mice were generated on C57BL/6J; *CK1δ*^{flox} (N>12), *Bmal1*^{flox} (N>9), *Vip-ires-Cre* (N>7), *Per2::Luc* (N>10), and *Rosa26-LSL-Cas9-2A-EGFP* (N>7) were backcrossed to C57BL/6J before use; *CaMKIIα-Cre* was backcrossed to C57BL/6N (N>10), then crossed to *CK1δ*^{flox} and *Per2::Luc*. We compared the conditional knockouts with controls whose genetic backgrounds were comparable."

We believe that our protocols meet the widely accepted standards of today.
