# Supplementary material for: In vivo recording of suprachiasmatic nucleus dynamics reveals a dominant role of arginine vasopressin neurons in circadian pacesetting
Source: PLoS Biol. 2023 Aug 29;21(8):e3002281. doi: 10.1371/journal.pbio.3002281 (PMC10465001; doi:10.1371/journal.pbio.3002281)
Supplement: Attachment — Submitted filename: Response to Reviewers.docx [file pbio.3002281.s007.docx]

**Response to Reviewers**

The authors would like to thank all reviewers for their valuable comments and suggestions, which guided us in significantly improving our manuscript. Our detailed responses to the reviewers’ concerns are itemized below.

**Responses to Reviewer #1**

*In this ms, the authors mainly attempted to explain the dominant role of AVP neurons in the regulation of the period of ensemble SCN by deleting CK1δ specifically in AVP, CaMKIIα, and VIP neurons in mice. The rhythm of locomotor activity and ex vivo PER2::LUC signals in the shell and core regions of SCN were recorded and analyzed. The rhythms of AVP and VIP neuronal [Ca^2+^]_i_  are also recorded in AVP-CK1δ KO mice. Although these data demonstrate that AVP neurons play a vital role in regulating the period of cellular circadian rhythm in vivo, more data are needed to demonstrate how AVP neurons regulate rhythm of SCN.*

**Response:** We appreciate the reviewer’s positive comments.

*Major concerns:*

*1	In this paper, the authors emphasized that AVP neurons regulate the period of cellular circadian rhythm of VIP neurons in vivo. However, further investigation of the relationships between AVP neurons and VIP neurons is needed. For example, the projection of AVP neurons to VIP neurons could be tested by retrograde or anterograde tracing. Also, it would be better to measure the [Ca^2+^]_i_ rhythm of VIP neurons with activation or inhibition of AVP neurons.*

**Response:** We thank valuable suggestions. We have confirmed that AVP neurons send projections, although not many, to VIP neurons by anterograde tracing (new Figure 7A-B). In addition, we have demonstrated in vivo that VIP neurons respond to optogenetic activation of AVP neurons by increasing [Ca^2+^]_i_ (new Figure 7C-F). As discussed in the manuscript, this effect of AVP neurons on VIP neurons may be direct or indirect (Text-revision, line 333). We plan to elucidate the exact mechanism of AVP→VIP neuron interaction as a step next to the current study. Also, Gizowski et al. recently reported that optogenetic stimulation of SCN AVP neurons advances the circadian behavior rhythm (Nature 2020, doi:10.1038/s41586-020-2471-x, ref 48). We have mentioned their finding in the discussion (Text-revision, line 330).

*2	It might be better to observe whether the period of behavioral rhythm is lengthened in AVP receptor knockout mice.*

**Response:** We selectively disrupted the *Avp* gene in SCN AVP neurons by using gRNA-Cas9 knockdown technique but observed no significant effect on the lengthened free-running period of *Avp-CK1δ^−/−^* mice (new Figure S5). This observation suggests that the cellular period of AVP neurons is transmitted to other SCN neurons by molecules other than AVP or by a redundant mechanism involving AVP. We have discussed the possible cases in the manuscript (Text-revision, line 355). We would like to consider the identification of such transmitters as a future step in this line of research.

*3	The author emphasizes that specific CK1δ deletion in AVP neurons is similar to CK1δ deletion in CaMKIIα neurons, so why not record AVP and VIP-neuronal [Ca^2+^]_i_ rhythm in CaMKIIα-CK1δ^−/−^ mice as positive control? And Vip-CK1δ^−/−^ mice as negative control.*

**Response:** Unfortunately, it is technically impossible to record AVP-neuronal [Ca^2+^]_i_ in *CaMKIIα-CK1δ^−/−^* or *Vip-CK1δ^−/−^* mice in the current condition. To express GCaMP in AVP neurons, we use *Avp-Cre* mice and Cre-dependent AAV vectors. When AAV is injected into the SCN of *CaMKIIα-CK1δ^−/−^*crossed with *Avp-Cre* mice, GCaMP would be expressed throughout the SCN. When injected to *Vip-CK1δ^−/−^* crossed with *Avp-Cre* mice, GCaMP would be expressed in both VIP and AVP neurons, and the mice are then *Avp&Vip-CK1δ^−/−^*. Thus, to express GCaMP expression in AVP neurons of *CaMKIIα-CK1δ^−/−^* or *Vip-CK1δ^−/−^* mice, we need to develop another AVP-neuron-specific driver mouse line using the Tet-system or FLP-FRT system. It is possible to record VIP-neuronal [Ca^2+^]_i_ in *CaMKIIα-CK1δ^−/−^* (by crossing with *Vip-tTA* line) or *Vip-CK1δ^−/−^* mice. However, we believe that such an experiment would not provide enough information to be worth the enormous effort of generating mice and long-term in vivo fiber photometry.

*4	The [Ca^2+^]_i_ rhythm of VIP neuron located in core SCN shows longer period in Avp-CK1δ^−/−^ mice while the period of core SCN in Avp-CK1δ^−/−^; Per2::Luc mice is similar to control, how to explain the inconsistency?*

**Response:** This observation is one of the essential points of this study. As discussed in the manuscript, slicing may damage the usual connections and humoral environment of the SCN so that AVP neurons cannot transmit period information to other SCN neurons. Several reports have reported similar inconsistencies, as mentioned in the Discussion (Text-revision, line 340, 364).

*5	According to the result of previously published in the Neuron (2015) and Current Biology (2016), AVP neurons played a vital role in the regulation of SCN rhythm, and deleting different genes result in different circadian phenotypes. Thus, why not explain the molecular mechanism of AVP neurons regulate circadian rhythm?*

**Response:** As described in our response to comment 2, we have investigated the involvement of the AVP peptide in the transmission of the AVP neuronal circadian period to behavior in vivo. However, the knockdown of AVP expression did not alter the free-running period of *Avp-CK1δ^−/−^* mice. We have previously reported that the lack of GABAergic transmission of AVP neurons also does not alter their behavioral period (Maejima et al., PNAS, 2021, ref 36). SCN AVP neurons express multiple neuropeptides in addition to AVP and GABA. Therefore, they may regulate the circadian rhythm by another peptidergic transmission or by a combination of several transmitters (Text-revision, line 360). Because the SCN explants do not recapitulate the lengthened behavior period of *Avp-CK1δ^−/−^* mice as discussed in comment 4, we cannot use them to efficiently screen for the candidate transmitters of the AVP neuronal circadian period. Nevertheless, we have demonstrated that AVP neurons activate VIP neurons in vivo, as described in our response to comment 1. We agree that understanding the molecular mechanism of AVP neuronal regulation of circadian rhythm, which seems complicated, should be the next critical issue to be addressed.

*6	Fig.3 and Fig.4 may be integrated into one figure.*

**Response:** We have integrated these two into Fig. 3.

*7	In P23, a spelling error of "P <0.01" should be "P < 0.01"?*

**Response:** We have corrected the spelling error.

**Responses to Reviewer #2**

*In this manuscript, the authors build on an earlier investigation in which they assessed how panSCN knockout of Ck1delta influence suprachiasmatic nuclei (SCN) and SCN-mediated behavioral rhythms in mice. They use standard approaches such wheel-running and ex vivo PER2::LUC bioluminescence measures as well as in vivo examination of calcium rhythms in the SCN via fibre photometry.  A range of genetic mouse models are employed and both male and female mice of these models are used.   The strength of the manuscript is the range of approaches, but the outcomes are not clear. The authors have hypothesized as to why there are disparities in the results from different experimental settings, but they do not test these.*

**Response:** We appreciate the reviewer’s valuable comments.

*1) The authors seem somewhat surprised that there are disparities between the period of behavioral rhythms and the period of the SCN (as determined ex vivo by measurement of rhythms in PER2::LUC). However, such differences have been documented for over 10 years (e.g. Hughes et al., PlosOne 2011) and indicate how difficult it can be to compare these two measures of circadian rhythms. In some instances, the periods are comparable, but in others (such as here), they are not. Clearly there are a lot of other factors are at play (such as does the knockout of CK1delta affect how well the SCN slice survives culturing, is the SCN reset by the culturing, etc which are not really considered by the authors).  It is notable that to demonstrate the importance of VIP to SCN PER2::LUC rhythms, Maywood and colleagues had to leave the SCN culture to damp out over 10+ days.*

**Response:** We fully agree with the reviewer’s point. Indeed, we recognize the previous important studies reporting the disparities between the period of behavioral rhythms and the period of the SCN determined ex vivo, as mentioned in the discussion (ref 63–66, Hughes et al., PlosOne 2011 has been added in the revised manuscript as ref 67). However, in the previous papers, the “in vivo period” mostly means the behavioral period, as the reviewer pointed out. Thus, there is a large gap between the SCN slice and behavioral rhythms. In this study, we directly measured the SCN dynamics in vivo to fill this gap. Notably, we performed such measurements in AVP and VIP neurons separately to examine the in vivo AVP→VIP neuron interaction, which has never been done before. Obviously, many factors, such as lower cell survival and resetting in culture, could cause the difference between in vivo and ex vivo. We have added this argument to the discussion (Text-revision, line 344). However, we do not aim to elucidate the exact mechanism of SCN explant rhythm in this study.

*2) Examination of the behavioral rhythms indicates that the wheel-running records are not being very carefully scrutinized.  In the supplemental Figure2, several examples of AVP-Bmal1^-/-^ are shown. The author conclude that most of these mice are arrhythmic. However, a number of them give evidence of 'splitting' ie that two bouts of behavior coincident with lighs-on and lights-off are sustained for 5-10 days in constant dark (1, 2, 3, 7, and 8) and in one example (3), these merge and to give at least the impression of longer period (ie >24h) rhythms. Thus, the conclusion that they are by and large arrhythmic is a generalization and is an over simplification.   This is important because the authors are relating behavioral activities to those of the SCN ex vivo.*

**Response:** We appreciate the reviewer’s critical comment. It is true that all *Avp-Bmal1^−/−^* mice maintained some rhythmicity in the wheel-running rhythm for some time after being released into DD. However, 7 out of 8 mice became arrhythmic after 10~20 days in DD. Such gradual dissipation of the wheel-running rhythm is a feature common to *Nms-Bmal1^−/−^* mice, which has been reported to be arrhythmic (Lee et al, Neuron 2015, ref 21). The periodograms shown in Figure S2 are generated with wheel-running activity for the last 7 days in DD. We have indicated the days used for analysis in the actograms with vertical lines (Figure S2C). We have also added some description of the phenotype in the Results and Discussion to avoid an oversimplification (Text-revision, line 150, 417). We do not claim that *Avp-Bmal1^−/−^* mice are completely arrhythmic, but we would like to emphasize that their circadian rhythm is disrupted as much as that of *Nms-Bmal1^−/−^* mice.

*3) The studies detailed in this manuscript used male and female mice. Are these balanced in the various studies ie are there equal numbers of male and female used?  This is important as the behavioral records in Figure S2 show clear sex differences (in the control animals, examples 2 and 3 are from female mice) and in the AVP-Bmal1^-/-^ examples 4 and 5 are from female mice. Since wheel-running suppresses nocturnal rodent SCN activity (Meijer group) then the in vivo calcium measurements should reveal this sex differences in either controls or experimental animals.   This needs to be analyzed and documented.*

**Response:** We have added information on the sex of individual mice used for all experiments in S1 Data and Figure S2C. The numbers of males and females were not exactly equal, but we think they were balanced. About the sex of mice for fiber photometry experiments, most were male mice, but one or two female mice in each group (male mice number, 5 out of 5 for AVP-GCaMP: Control, 5 out of 6 for AVP-GCaMP: *Avp-CK1δ^−/−^*, 4 out of 6 for VIP-GCaMP: Control, 3 out of 4 for VIP-GCaMP: *Avp-CK1δ^−/−^*). Please understand that we used highly complex breeding, so it is difficult to obtain enough mice for experiments with males only. Also, except for Figure S2, we recorded home-cage activity to measure the behavioral rhythm, which shows fewer sex differences than the wheel-running. As the reviewer pointed out, the wheel-running activity rhythms in Figure S2C show obvious sex differences. However, both male and female *Avp-Bmal1^−/−^* mice became almost arrhythmic after prolonged housing in DD. We have stated in "Materials and Methods, Animals" that "whether we pooled data from both sexes or analyzed them separately, the conclusions we reached remained the same." (Text-revision, line 615)

**Responses to Reviewer #3**

*This is a very elegant and nicely conducted study with high experimental rigor. The authors replicate prior work that deletion of CK1delta in AVP neurons of the SCN lengthens the behavioral rhythm period (home cage activity). Here, they extend this finding by adding additional comparisons of wheel running data to CK1delta deletion throughout the SCN or just in VIP neurons which did and did not lengthen behavior period. Analysis of PER2::LUC explant rhythms from these same genotypes revealed a period lengthening effect in the SCN shell but only in the first cycle, as previously reported. The most novel contribution is that of the heroic in vivo calcium imaging of the AVP-CK1delta KO mice in both AVP and VIP neurons with simultaneous measurement of behavioral rhythms. These data showed that both populations lengthen period in vivo in phase with the lengthened behavioral rhythm. The results support the idea that AVP neurons are important for determining the period length of SCN rhythms in vivo but that cellular molecular clocks of AVP neurons require input from other SCN neurons (or other brain regions, as in the in vivo case). Some of these ideas have been supported before in the literature (although not all are recognized or discussed in the manuscript). Additional strengths of the study include the high rigor in methodology and statistical analysis. However, the impact of the paper could be increased by the authors addressing the following concerns:*

**Response:** We thank the reviewer’s encouraging comments.

*1.	The issue of AVP/VIP core/shell signaling has been addressed in other studies, including findings that AVP signaling modulates SCN period and phase in a spatially specific manner (PMID: 29931690) and that AVP receptor antagonists reduce PER2::LUC amplitude (PMID: 27626074 and PMID: 32460660). Not only are these papers not discussed but there seems to be a missed opportunity for simple pharmacology experiments in these sophisticated genetic manipulations, especially given that much of the PER2::LUC results were known from the prior publication.*

**Response:** Based on the reviewer's suggestion regarding the importance of the AVP peptide, we examined whether eliminating AVP expression from SCN AVP neurons shortens the behavioral free-running period of *Avp-CK1δ^−/−^* mice (new Figure S5). However, we did not observe a significant change. Therefore, the cellular period of AVP neurons may be transmitted to other SCN neurons by molecules other than AVP or by a redundant mechanism involving AVP. We have also discussed what has been reported concerning the role of AVP in SCN explants (Text-revision, line 356). Because PER2::LUC rhythms in SCN explants of *Avp-CK1δ^−/−^* mice rapidly lost period-lengthening, pharmacological V1a blockade experiments may not provide much useful information. As a next step, we plan to investigate the molecular mechanism by which AVP neurons transmit their cellular period to other SCN cells.

*2.	The Summary states that the rhythms of these mice "did not recapitulate the period lengthening …" which is a bit overstated given that the period of the first cycle of shell neurons was substantially lengthened (as previously published).*

**Response:** We have modified the sentence to avoid an overstatement ((Text-revision, line 31, 158).

*3.	Throughout the paper there is this tendency to compare period effects among the three methods (two in vivo and one ex vivo; see direct statements on the bottom of page 13) - however, it is important to note that there are very important methodological differences in how period is calculated. For example, in the wheel running behavior, period is calculated on days 10-24 in DD. The explants however, come straight out of LD and period is really estimated one cycle at a time. So, this would be akin to measuring the activity onset to onset on the first day of DD, and so on. This period estimation has a similar problem for the calcium imaging which is also calculated differently (days 8-10 in DD). If the goal is to directly compare in vivo to ex vivo then maybe a missing comparison in the study is to measure GCaMP in the ex vivo condition (just like PER2::LUC) to then see if the results are similar. This would also address the issue of whether the seemingly disparate results are due to a focus on the TTFL output (PER2::LUC) versus a physiological output (calcium oscillations, indicative of neuronal activity), which can also be dissociated. In all, these are important things to consider before concluding that the "SCN slice data may not necessarily be relied upon to support arguments …"*

**Response:** We agree with the reviewer’s suggestion that measuring GCaMP in the ex vivo condition would allow us to directly compare in vivo and ex vivo dynamics of the SCN network. Indeed, we have previously tried to monitor the GCaMP signal rhythm in SCN slices prepared from adult mice in collaboration with Dr. Ryosuke Enoki, who has made many beautiful recordings of GCaMP rhythms in neonatal organotypic SCN cultures. However, a similar protocol did not work for adult SCN slices. We failed to record stable rhythms due to the continuous distortion of SCN slices, possibly caused by phototoxicity. Consistently, we are not aware of any previous reports that succeeded in recording Ca rhythms in adult SCN slices. So instead, we calculated the day-by-day change of periods of in vivo Ca rhythms in DD (new Figure 6H-I). As a result, we confirmed that the periods of both AVP and VIP Ca rhythms were stably lengthened in vivo. Regarding the dissociation of TTFL and Ca rhythms, it is unlikely that these two oscillate with different periods. We have added these arguments in the Discussion (Text-revision, line 346). We have also deleted the sentence “Thus, SCN slice data may not necessarily be relied upon to support arguments about the organization and inter-regional communication.”
